# WY-14643 attenuates lipid deposition via activation of the PPARα/CPT1A axis by targeting Gly335 to inhibit cell proliferation and migration in ccRCC

**DOI:** 10.1186/s12944-022-01726-7

**Published:** 2022-11-16

**Authors:** Rui Wang, Jun Zhao, Jiacheng Jin, Yun Tian, Lan Lan, Xuejian Wang, Liang Zhu, Jianbo Wang

**Affiliations:** 1 Department of Urology, The First Affiliated Hospital of Dalian Medical University, Dalian, China; 2 Department of Urology, The Third People’s Hospital of Chengdu, Chengdu, China; 3 College of Basic Medical Science, Dalian Medical University, Dalian, China

**Keywords:** ccRCC, WY-14643, Lipid accumulation, PPARα, CPT1A

## Abstract

**Background:**

Histologically, cytoplasmic deposits of lipids and glycogen are common in clear cell renal cell carcinoma (ccRCC). Owing to the significance of lipid deposition in ccRCC, numerous trials targeting lipid metabolism have shown certain therapeutic potential. The agonism of peroxisome proliferator-activated receptor-α (PPARα) via ligands, including WY-14,643, has been considered a promising intervention for cancers.

**Methods:**

First, the effects of WY-14,643 on malignant behaviors were investigated in ccRCC in vitro. After RNA sequencing, the changes in lipid metabolism, especially neutral lipids and glycerol, were further evaluated. Finally, the underlying mechanisms were revealed.

**Results:**

Phenotypically, the proliferation and migration of ccRCC cells treated with WY-14,643 were significantly inhibited in vitro. A theoretical functional mechanism was proposed in ccRCC: WY-14,643 mediates lipid consumption by recognizing carnitine palmitoyltransferase 1 A (CPT1A). Activation of PPARα using WY-14,643 reduces lipid deposition by increasing the CPT1A level, which also suppresses the NF-κB signaling pathway. Spatially, WY-14,643 binds and activates PPARα by targeting Gly335.

**Conclusion:**

Overall, WY-14,643 suppresses the biological behaviors of ccRCC in terms of cell proliferation, migration, and cell cycle arrest. Furthermore, its anticancer properties are mediated by the inhibition of lipid accumulation, at least in part, through the PPARα/CPT1A axis by targeting Gly335, as part of the process, NF-κB signaling is also suppressed. Pharmacological activation of PPARα might offer a new treatment option for ccRCC.

**Supplementary Information:**

The online version contains supplementary material available at 10.1186/s12944-022-01726-7.

## Introduction

At least 400 thousand people are identified as renal cell carcinoma (RCC) each year in the world, to which approximately half of the cases succumb [1]. Although the growing number of studies focus on diagnosing and treating kidney cancer, there is still a high mortality rate [2]; approximately 70% of the cases is clear cell renal cell carcinoma (ccRCC) [3]. Up to 30% ccRCC patients have a poor prognosis, even if they are caught early and treated with surgical methods [4]. The prognosis of these patients remains unsatisfactory [5].

Accumulating evidence has indicated that aberrant metabolism contributes to the growth and metastasis of tumors [6]. Moreover, recent studies have identified lipid metabolism reprogramming as a trait of cancer [7]. Especially in the cytoplasm of ccRCC cells, a large amount of lipids and glycogen components accumulate, tumor tissues show a characteristic clear cytoplasm on pathological sections, which is closely linked to the prominent metabolic disorder of ccRCC [8]. Because of alterations in multiple metabolic pathways, ccRCC is increasingly recognized as a “metabolic disease” [9]. In ccRCC cells, due to Von Hippel‒Lindau (VHL) gene mutation, hypoxia-inducible factors (HIFs) accumulate, and the Warburg effect occurs, resulting in increased synthesis of glycogen and fatty acids [10, 11]. Thus, targeting lipid biosynthesis may be a novel strategy to manage the development of ccRCC [12].

WY-14,643 was recognized as a lipid-lowering agent [13]. Currently, WY-14,643 is universally acknowledged as a peroxisome proliferator-activated receptor α (PPARα) agonist affecting lipid metabolism [14, 15]. PPARα promotes fatty acid oxidation and glycogen synthesis while inhibiting lipogenesis and glycolysis [16]. Due to its position at the intersection of metabolism and cellular processes [17], PPARα has drawn attention as a potential target for malignancies, such as colorectal cancer and hepatocellular carcinoma [18–22]. It remains unclear, however, whether WY-14,643 is effective in treating ccRCC and its anticancer mechanism.

When mitochondrial oxidative phosphorylation occurs, carnitine palmitoyltransferase 1 A (CPT1A) facilitates the translocation of acyl groups, thus acting as a pivotal rate-limiting enzyme [23]. The critical role of fatty acid oxidation (FAO) in cancer metabolism has led to renewed interest in CPT1A as a pivotal mediator [24]. As a key factor in lipid metabolism, CPT1A is engaged in the management of lipid metabolism through FAO in colorectal cancer [25], breast cancer [26], nasopharyngeal carcinoma [27], etc. Recently, emerging evidence has shown CPT1A is downregulated in ccRCC, concomitant with suppressed FAO; at the same time, overexpression of CPT1A reduces lipid accumulation in ccRCC, thus suppressing cell proliferation [28, 29]. In addition, in ccRCC cells, lipid droplets are synthesized only when CPT1A (regulated directly by HIF) is repressed [30]. As CPT1A has become a core regulator in lipid metabolism in ccRCC, therapies targeting CPT1A might have great potential for ccRCC treatment.

Since downregulation of PPARα significantly suppresses CPT1A expression and subsequently suppresses FAO in melanoma cells [31], the hypothesis that activation of PPARα by WY-14,643 might affect CPT1A was raised. To test this hypothesis, the impacts of WY-14,643 on malignant phenotypes were examined in ccRCC, and possible underlying mechanisms were also detected. In the current study, it was demonstrated that WY-14,643 activates PPARα by targeting Gly335 and then upregulates CPT1A to reduce lipid deposition, as part of the process, NF-κB signaling is also suppressed.

## Methods

### Chemicals and antibodies

WY-14,643 was sourced from ApexBio (USA). GW6471 was obtained from Topscience (China). Dimethyl sulfoxide (DMSO) was used as the solvent and was diluted to < 0.1% before use. Antibodies specific to GAPDH, CDK2, Cyclin A, Cyclin B1, Vimentin, CPT1A, Cleaved caspase-3, Cleaved PARP, Bax and Bcl-2 were purchased from Proteintech Group (China). Antibodies specific to N-cadherin, NF-κB pathway were purchased from Cell Signaling Technology (USA). Antibodies specific to Caspase-3 were purchased from Abcam (England).

### Cell culture

The human renal cell carcinoma cell lines 786-O and A498 and the human proximal tubular cell line HK2 were sourced from the American Type Culture Collection (USA). Cells were grown in a sterile incubator 37 °C containing 5% CO_2_. All the cells were grown in the recommended medium containing 1% penicillin/streptomycin and 10% fetal bovine serum (Gibco, USA).

### Colony formation assay

In a 6-well plate, 300 ccRCC cells per well were inoculated after administration of WY-14,643 for 24 h. When colonies were formed and large enough to be visualized (nearly 9 days), the cells were labeled with 0.1% crystal violet after fixation in formaldehyde. Finally, the colonies were counted.

### Cell viability assay

In a 96-well plate, after 2*10^3^ ccRCC cells seeded per well adhered, different doses of WY-14,643 were administered for specific hours. Each well was filled with 10 µl of CCK-8 (ApexBio, USA) reagent. After 1.5 h, the OD values at 450 nm were obtained with a microplate reader.

### Wound healing assay

Briefly, in a six-well plate, ccRCC cells were cultured until confluence was observed. After serum starvation for 6 h, a scratch was made. Following the removal of detached cells and debris with PBS, appropriate doses of WY-14,643 were applied. Images were taken at 0 and 12 h after observing the wound gap. Cell migration percentages were calculated by ImageJ.

### Transwell assay

24-well plates (Corning, USA) containing 8 μm membrane filters were used for the assays. After administration of specified concentrations of WY-14,643 for 24 h, 1*10^4^ ccRCC cells were seeded per well. Twenty-four hours later, the bottom cells were stained after removing the upper cells. Cell migration percentages were calculated by ImageJ.

### Oil red O (ORO) staining

To determine lipid droplets, after fixation in 10% formaldehyde, ccRCC cells were rinsed for 5 min in 60% isopropanol. Following staining for 10 min with freshly prepared ORO (Solarbio, China), images were taken under a microscope (Leica, Germany). ORO quantification was performed by adding 300 µl of isopropanol to the dried cells, incubating for 3 min, and determining the absorbance at 490 nm.

### Nile red staining

To quantify the intracellular neutral lipid content, 1 µg/ml Nile red fluorescent stain was used to label ccRCC cells (ApexBio, USA). Briefly, 1*10^4^ cells per well were seeded and inoculated. After treatment with different doses of WY-14,643, the cells were rinsed with Triton X-100. Later, intracellular neutral lipid deposits were observed by staining with Nile red, and nuclei were identified by Hoechst 33,342 (Beyotime, China). Images were then captured.

### Total glycerol quantification test

Following WY-14,643 treatment, total glycerol content was examined by a cell glycerol enzymatic assay kit (E1012, Applygen, China). Briefly, after the cells were trypsinized, centrifuged, and lysed, and lipase was inactivated, then the absorbance at 550 nm was detected after 10 µl supernatant and 190 µl working solution were preserved in 37 °C water bath for 10 min. Finally, the total glycerol content was normalized to the total protein amount (per mg units).

### Western blotting analysis

After extracting total protein and determining protein concentration, the SDS PAGE gels loaded with 30 µg of protein samples were transferred to polyvinylidene difluoride membranes in an ice-water bath. Incubation of membranes with diluted primary antibodies was performed following the blocking procedure. Next day, after 1.5 h-incubation in secondary antibodies, the membranes were exposed to the Bio-Rad ChemiDoc system followed by 3 min of reaction in the ECL mix.

### Reverse transcription-quantitative polymerase chain reaction (RT‒qPCR)

After extracting total mRNA and determining the concentration, cDNA was synthesized using the HiScript Q RT SuperMix for qPCR kit (Vazyme, China). Subsequently, water, primers, ChamQ Universal SYBR qPCR Master Mix (Vazyme, China), and cDNA templates were mixed in a Bio-Rad Real-Time PCR Detection System according to the protocols. The following is a list of primer sequences used: CPT1A, forward 5′-TTCAGTTCACGGTCACTCCG-3′, reverse 5′-TGACCACGTTCTTCGTCTGG-3′; GAPDH, forward 5′-GCACCGTCAAGGCTGAGAAC-3′, reverse 5′-TGGTGAAGACGCCAGTGGA-3′.

### Flow cytometry

After treatment with specified concentrations of WY-14,643 for 48 h, 1*10^6^ 786-O cells were fixed in ethanol overnight at -20 ℃. The next day, after the fixed cells were centrifuged and washed, Triton X-100 and RNase were added. Finally, propidium iodide was diluted to 100 µg/ml. After that, cell cycle distribution was analyzed by BD flow cytometry.

### Bioinformatics analysis

Next-generation transcriptomic sequencing was conducted by the Illumina NovaSeq 6000 sequencing platform (Biozeron, China). To further analyze related pathways, Gene Ontology (GO) and Kyoto Encyclopedia of Genes and Genomes (KEGG) were both calculated by GOATOOLS and KOBAS. Protein‒protein interactions (PPIs) were estimated by The Search Tool for the Retrieval of Interacting Genes (STRING; http://string.embl.de). To investigate the correlation between PPARα and CPT1A, gene correlation analysis was conducted by Gene Expression Profiling Interactive Analysis (GEPIA) and The University of Alabama at Birmingham Cancer Data Analysis Portal (UALCAN).

### Molecular modeling

Whether WY-14,643 binds specifically to the PPARα protein was investigated by molecular docking. The protein receptor was accessed from the RCSB PDB protein structure database (https://www.rcsb.org/). To optimize WY-14,643, the Polak-Ribière-Polyak conjugate gradient algorithm was used along with the semiempirical PM3 method. Semiflexible docking between the ligand and the receptor was performed via AutoDockTools using the AutoDock Vina program. The docking score of the ligand molecule WY-14,643 and the protein receptor PPARα was − 7.5 kcal/mol. Finally, the results were visualized using Ligplot and PyMOL software.

### Statistical analysis

All value data were analyzed by SPSS 11.0 and presented as the mean ± SD. Differences in groups were assessed by Student’s t test or one-way ANOVA. *P* < 0.05 was taken into consideration for the significance.

## Results

### WY-14,643 inhibits ccRCC cell proliferation and changes the morphology

**Fig. 1:**
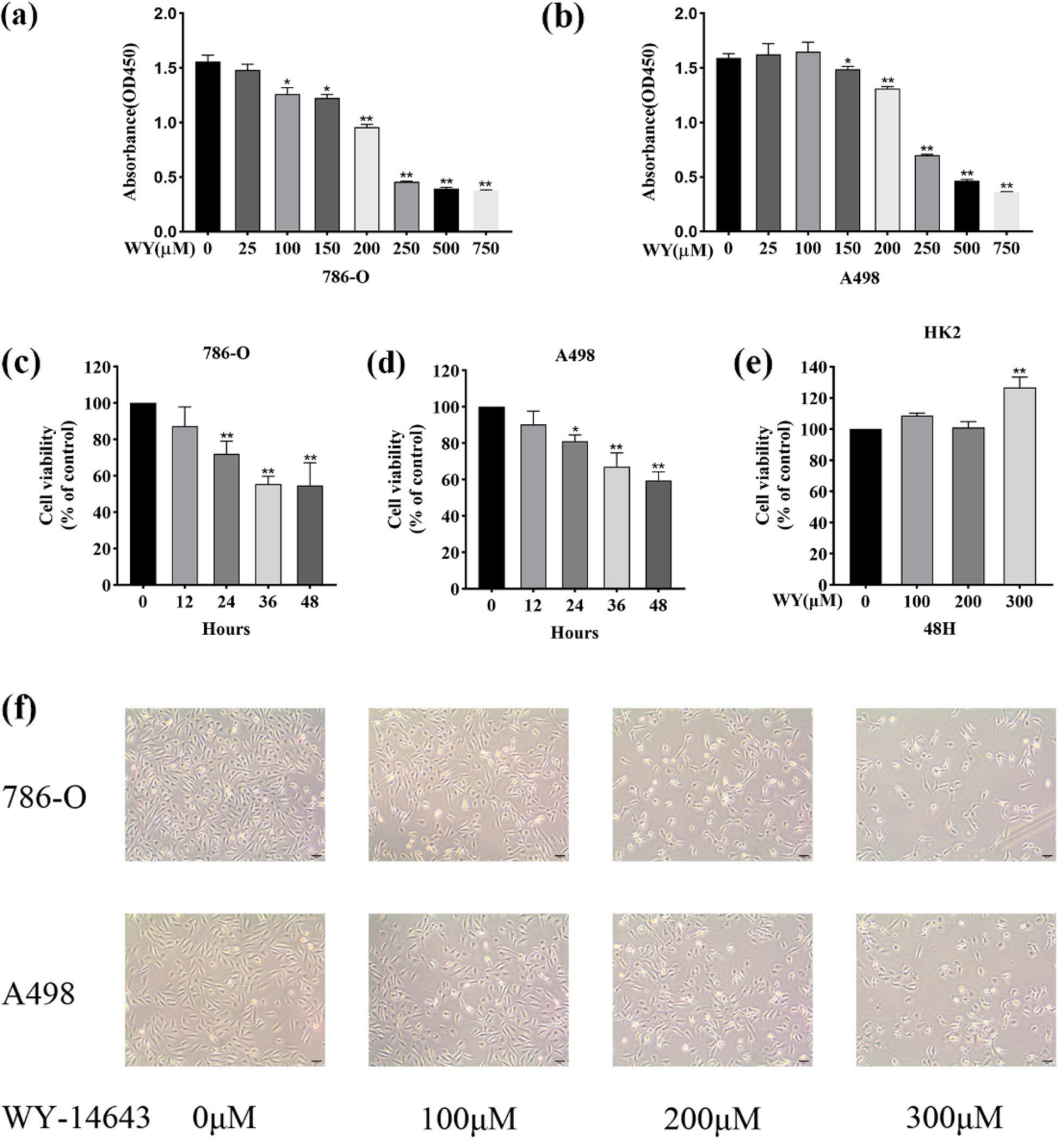
WY-14,643 inhibits ccRCC cell proliferation and changes the morphology. **a** and **b.** 48 h after treatment with WY-14,643 at the indicated doses, the absorbance of ccRCC cells was examined at 450 nm. **c** and **d**., cell viability was calculated at the indicated time points after 200 µM WY-14,643 applied to ccRCC cells. **e**. HK2 cell viability was determined after specified doses of WY-14,643 were administered for 48 h. **f**. The changes in cell morphology were observed after administration of WY-14,643 for 48 h and photographed under an inverted microscope. Magnification, 100x. *P < 0.05, **P < 0.01. WY, WY-14,643

Cell proliferation was examined after treatment with WY-14,643, further morphological changes were also observed. Figure 1a and b represent that following treatment with increasing doses of WY-14,643, 786-O and A498 cells show a decreased viability. The half maximal inhibitory concentration (IC50) values were 219.1 ± 11.7 µM and 207.4 ± 8.3 µM for 786-O and A498 cells, respectively. Figure 1c and d show that cell viability decreased significantly with time after 200 µM WY-14,643 were applied to 786-O and A498 cells. After WY-14,643 treatment, proximal tubular cells were evaluated for viability, a CCK-8 assay using HK2 cells was next performed. According to Fig. 1e, pretreatment with WY-14,643 did not affect HK2 cell viability at specified concentration (from 0 µM to 300 µM) for 48 h. Finally, it was observed that WY-14,643-treated cells exhibited reducer cell‒cell contact and fewer filopodia versus control (0 µM)-treated cells, as shown in Fig. 1f. These results demonstrated that WY-14,643 inhibits ccRCC cells proliferation dose- and time-dependently and WY-14,643 administration suppresses cell‒cell contact and filopodia formation.

### WY-14,643 impairs the colony formation of ccRCC cells and induces cell cycle arrest

**Fig. 2:**
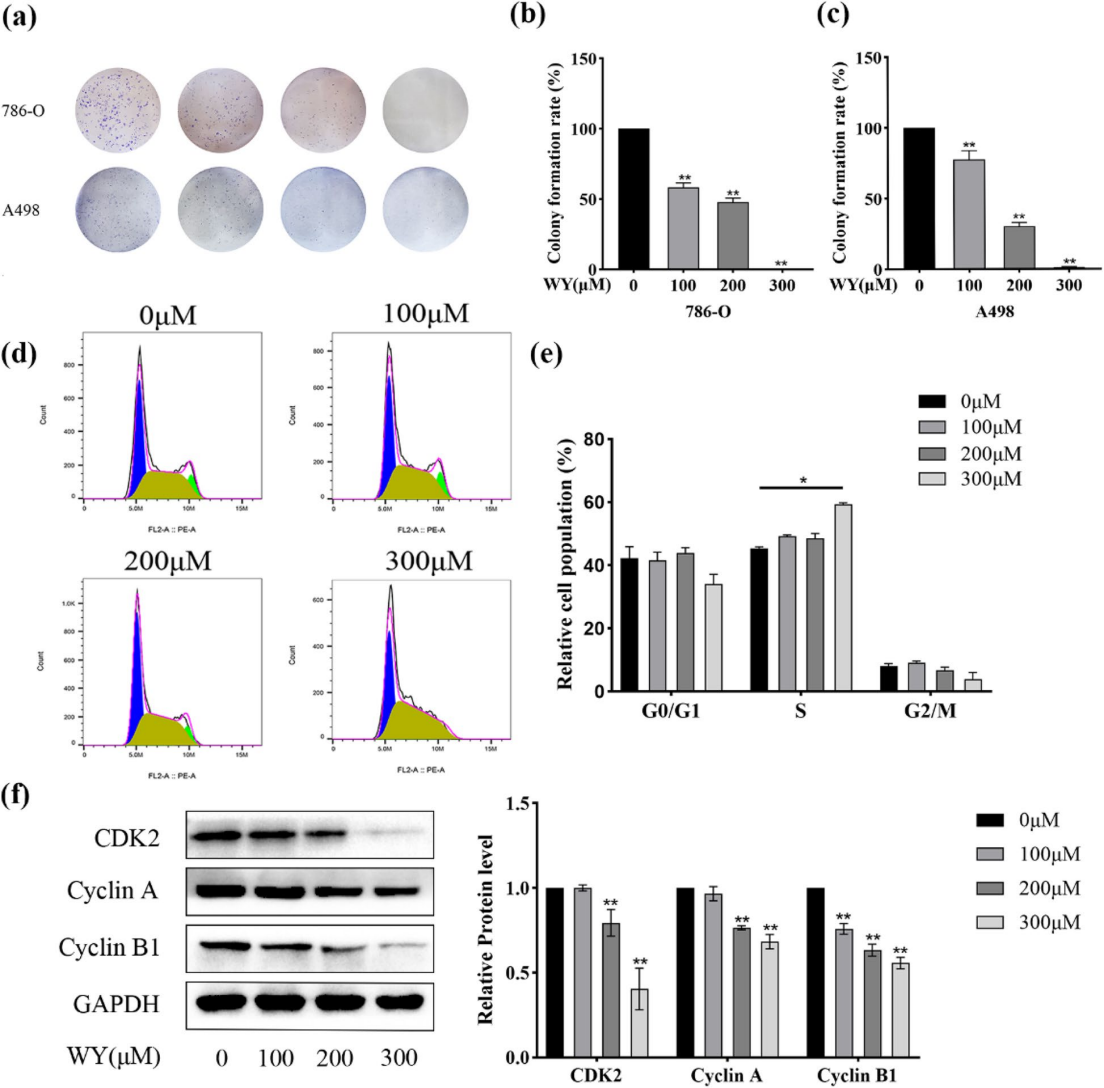
WY-14,643 impairs the colony formation of ccRCC cells and induces cell cycle arrest. **a**. Images of colony formation were captured after pretreatment with WY-14,643 in ccRCC cells. **b** and **c**. The colony formation rate was analyzed. **d**. An examination of the cell cycle distribution was performed in 786-O cells after specified doses of WY-14,643 were applied for 48 by BD Accuri C6 flow cytometer. **e**. Cell cycle distribution was quantified. **f**. After WY-14,643 was administered for 48 h, S-G2/M-related proteins were detected and analyzed quantitatively (*n* = 3). *P < 0.05, **P < 0.01. WY, WY-14,643

The colony formation assay was subsequently conducted. Figure 2a-c illustrates that treatment with WY-14,643 significantly impaired colony formation ability and decreased the colony formation rate when compared to that in the control groups. Cell cycle changes are usually accompanied by changes in cell proliferation. Thus, cell cycle changes were evaluated by flow cytometry after WY-14,643 treatment. WY-14,643 treatment of 786-O cells for 48 h dose-dependently led to cell retention in the S phase (Fig. 2d), and the data in Fig. 2e clearly show that there was an increase from 45.4 to 59.4% of 786-O cells in the S phase. The results of the experiment were further confirmed by western blotting following treatment with WY-14,643, which showed that the S-G2/M-related proteins CDK2, cyclin A, and cyclin B1 were downregulated in 786-O cells after WY-14,643 treatment (Fig. 2f). These data provide evidence that S phase arrest contributes to the antiproliferative effects of WY-14,643, at least in part. In summary, WY-14,643 effectively inhibits the growth of ccRCC.

### WY-14,643 suppresses ccRCC cell migration

**Fig. 3:**
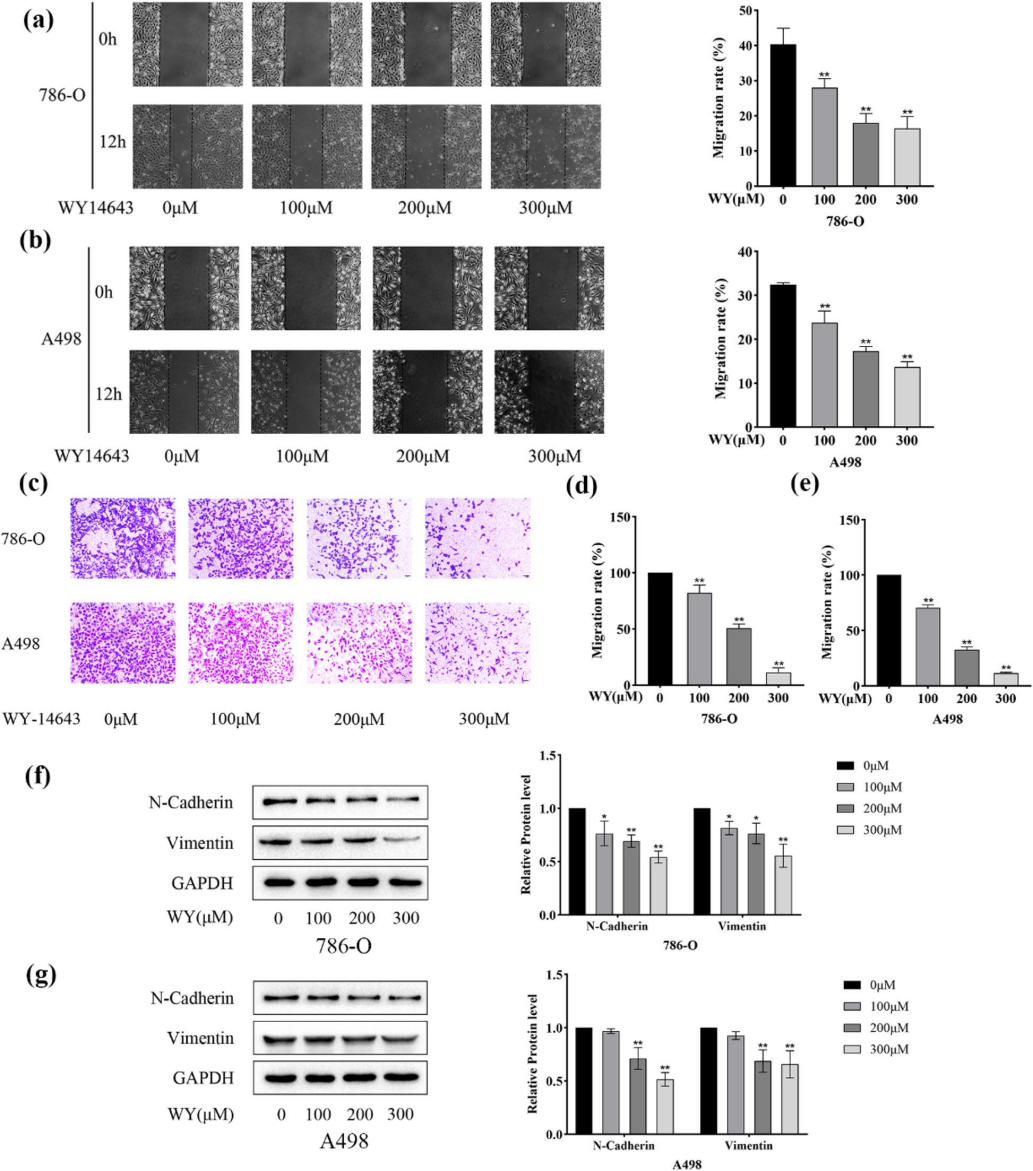
WY-14,643 suppresses ccRCC cell migration. **a.** Photographs of wound gaps were taken 12 h after scratching (left) and cell migration percentages were calculated (right) in 786-O cells. **b.** In A498 cells, photographs of wound gaps were taken 12 h after scratching (left) and cell migration percentages were calculated (right). **c**. After pretreatment with WY-14,643, a Transwell assay was conducted to assess cell migration and images were taken. Magnification, 100x. **d** and **e**. Cell migration percentages were calculated. **f** and **g**. After treatment with various doses of WY-14,643, mesenchymal markers were detected by western blotting and analyzed quantitatively (*n* = 3). *P < 0.05, **P < 0.01. WY, WY-14,643

Transwell assays and wound healing assays were both performed for assessing cell motility. As Fig. 3a and b show, administration of WY-14,643 significantly suppressed the migration abilities of ccRCC cells compared with those of the control-treated cells. Cell migration was also quantitatively analyzed. In the Transwell assay, similar phenotypes were observed. Compared with the control, high WY-14,643 concentrations, especially 200 and 300 µM, significantly reduced migrating ccRCC cells (Fig. 3c). Results of quantitative analyses of the Transwell assay are shown in Fig. 3d and e. Cell motility and tumor metastasis are enhanced by epithelial-mesenchymal transition (EMT) [32]. To determine the possible mechanisms by which WY-14,643 affects cell migration, important mesenchymal markers were detected in 786-O cells. As Fig. 3f shows, treatment with WY-14,643 significantly downregulated N-cadherin and vimentin. In Fig. 3g, analogous results are shown in A498 cells. According to these results, EMT inhibition might account for the inhibition of cell migration by WY-14,643.

### WY-14,643 treatment disrupts abnormal lipid metabolism in ccRCC cells

**Fig. 4:**
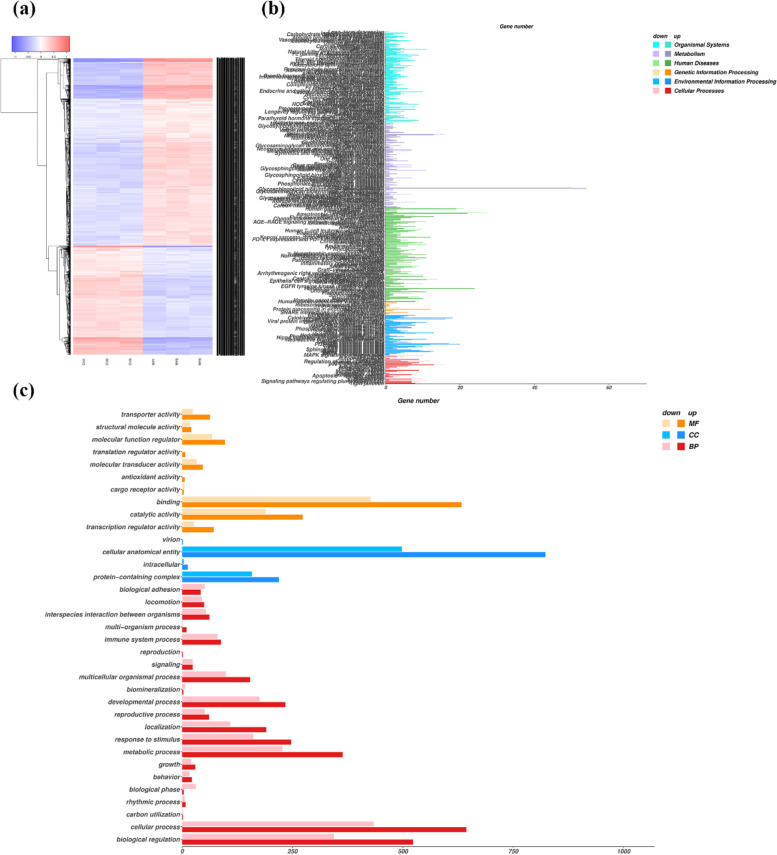
Many genes were considered related to metabolism or metabolic processes based on transcriptomic sequencing and subsequent annotation. **a**. Heatmap visualization of differential expression between the WY-14,643 treatment group and the ctrl groups. **b**. KEGG annotations of the DEGs. The layer 1 pathways are marked in the top right-hand corner. **c.** GO annotations of the DEGs. The abscissas in **a** and **b** represent the number of genes annotated to a certain pathway

Next-generation transcriptomic sequencing was conducted to investigate the differentially expressed genes (DEGs) and enriched pathways in depth in ccRCC cells after treatment with WY-14,643. As shown in Fig. 4a, global differential gene expression clustering analysis was conducted based on FPKM counts, and 1075 upregulated genes and 625 downregulated transcripts were identified. As shown in Supplementary Fig. 1a and b, KEGG pathway analysis and GO term enrichment analysis revealed crucial genes and pathways, including p53 signaling pathway, Hippo signaling pathway, movement, retinol metabolism, drug metabolism, apoptosis, etc. These results indicated that WY-14,643 is worthy of exploration and expectation because it interfered with many biological and cellular processes, including development and differentiation, apoptosis, movement, etc. Then, as shown in Fig. 4b and c, according to KEGG and GO annotation, the maximum proportion of genes was annotated to metabolism pathways or metabolic processes, mainly including lipid metabolism, energy metabolism, catalytic activity, etc. Considering abnormal lipid metabolism in ccRCC and the lipid-lowering effect of WY-14,643, the role WY-14,643 in lipid metabolism in ccRCC was focused on. Accordingly, protein-coding genes correlated with lipid metabolism, especially triglyceride turnover, fatty acid activation and oxidation, were further identified. In addition, their expression levels were visualized with a heatmap (Supplementary Fig. 1c). The regulatory effect of WY-14,643 on triglyceride turnover and fatty acid oxidation in ccRCC can be accessed intuitively through the heatmap. All the results above suggest that WY-14,643 treatment disrupts abnormal lipid metabolism in ccRCC cells.

### WY-14,643 alleviates lipid accumulation in ccRCC cells

**Fig. 5:**
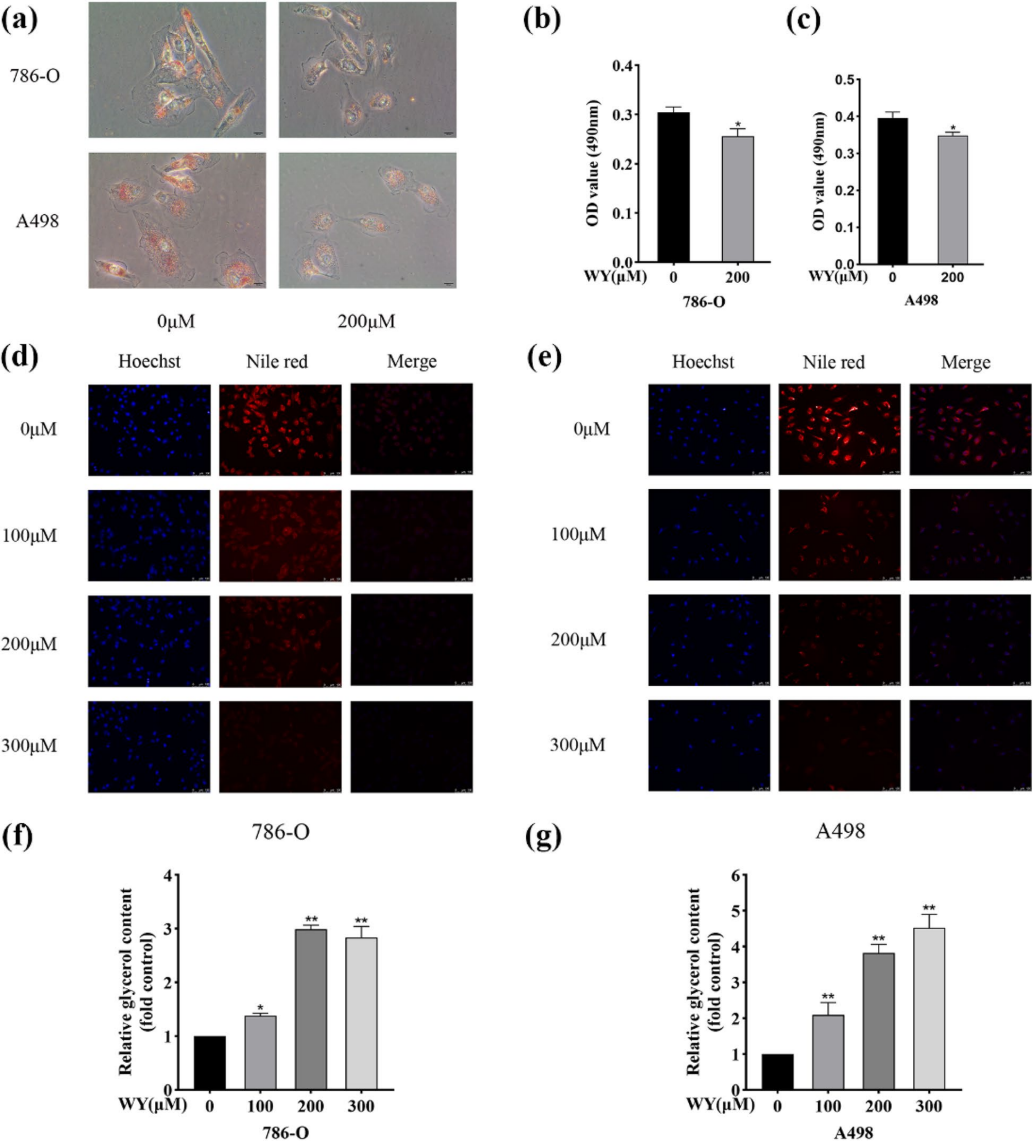
WY-14,643 facilitates triglyceride consumption in ccRCC cells. **a**. After 48 h-treatment with 200 µM WY-14,643, lipid droplets in ccRCC cells were evaluated by Oil red O staining. Magnification, 400x. **b** and **c**. The OD values at 490 nm were quantitatively measured. **d** and **e**. Neutral lipid levels were evaluated by Nile red staining after treatment with WY-14,643 at the indicated doses for 48 h, and images were captured. Magnification, 200x. **f** and **g**. The intracellular glycerol content was measured after 48 h-treatment with WY-14,643 at the indicated doses in 786-O and A498 cells. *P < 0.05, **P < 0.01. WY, WY-14,643

It is generally acknowledged that intracellular lipid accumulation is a significant characteristic of ccRCC. The effect of WY-14,643 on lipid metabolism was next investigated. As Fig. 5a represents, ccRCC cells stained with Oil red O displayed fewer lipid droplets after WY-14,643 treatment, and the absorbance was quantitatively examined, as shown in Fig. 5b and c. Nile red is a fluorescent stain specific for the detection of intracellular neutral lipids, and intracellular lipid levels were then identified by using Nile red staining. Figure 5d shows that WY-14,643 treatment obviously reduced intracellular neutral lipid levels in 786-O cells, especially at a high dose. A498 cells also showed similar results (Fig. 5e). The end products of triglyceride breakdown in the fat mobilization pathway are glycerol and fatty acids; here, the content of intracellular glycerol was further detected by using a glycerol content assay kit. The intracellular glycerol content increased with increasing concentrations of WY-14,643 treatment in ccRCC cells, as shown in Fig. 5f, g. The above results implied that WY-14,643 treatment effectively ameliorated lipid accumulation by facilitating triglyceride consumption and then increasing the level of glycerol.

### WY-14,643 inhibits lipid accumulation via the regulation of CPT1A in ccRCC cells

**Fig. 6:**
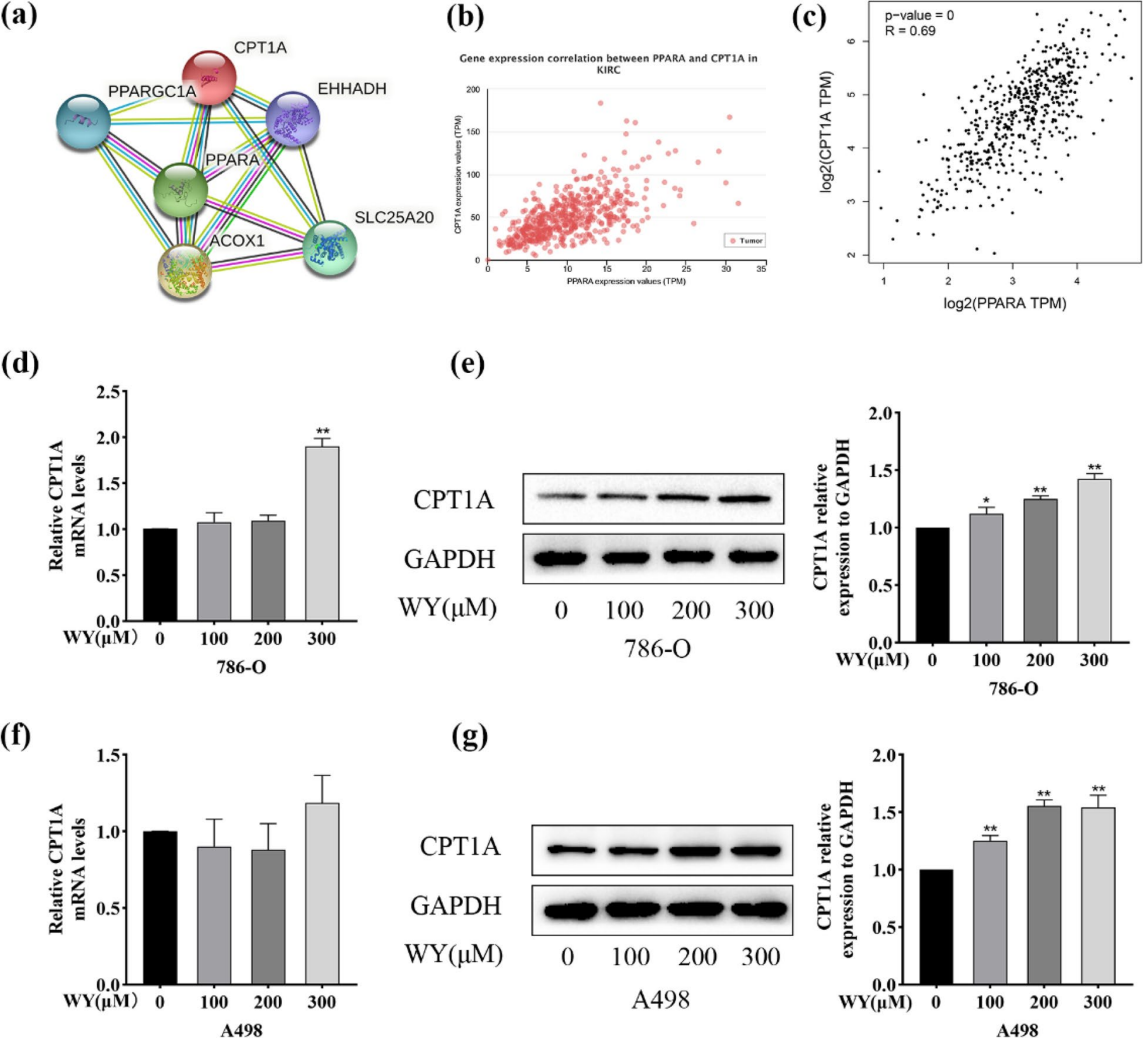
WY-14,643 upregulates CPT1A in ccRCC cells. **a**. PPI evaluated by STRING. **b** and **c**. In ccRCC, gene correlation analysis between PPARα and CPT1A through UALCAN and GEPIA. **d**. Specific doses of WY-14,643 were administered, and relative CPT1A mRNA expression levels were analyzed in 786-O cells (*n* = 3). **e**. Specified doses of WY-14,643 were treated, and CPT1A protein expression levels were detected and analyzed quantitatively in 786-O cells (*n* = 3). **f**. Different concentrations of WY-14,643 were applied, and relative CPT1A mRNA expression levels were analyzed in A498 cells (*n* = 3). **g**. Different concentrations of WY-14,643 were administered, and CPT1A protein expression levels were detected and analyzed quantitatively in A498 cells (*n* = 3). *P < 0.05, **P < 0.01. WY, WY-14,643

Multiple lines of evidence show that CPT1A, a core regulator in FAO in ccRCC, is bound and repressed by HIFs, resulting in the storage of fatty acids in lipid droplets, thus inducing a clear cytoplasmic phenotype [28–30]. Using STRING, the interplay between PPARα and CPT1A proteins was discovered in ccRCC, as shown in Fig. 6a. In addition, by UALCAN and GEPIA gene correlation analysis in ccRCC, the related coefficients of PPARα and CPT1A were *R* = 0.62 and *R* = 0.69, respectively (Fig. 6b and c). Both CPT1A mRNA and CPT1A protein were then evaluated in the current research. CPT1A mRNA levels did not change significantly with increasing WY-14,643 concentration, except for a slight increase when a high dose was administered to 786-O cells, as demonstrated in Fig. 6 d f. However, according to Fig. 6e g, the CPT1A protein levels were obviously elevated as the WY-14,643 concentration increased in ccRCC cells. CPT1A protein levels were consistent with the lipid droplet changes in Fig. 5a-e. These results implied that WY-14,643 treatment inhibited lipid accumulation via the regulation of CPT1A.

### WY-14,643 treatment reduces lipid deposition via the PPARα/CPT1A axis in ccRCC cells

**Fig. 7:**
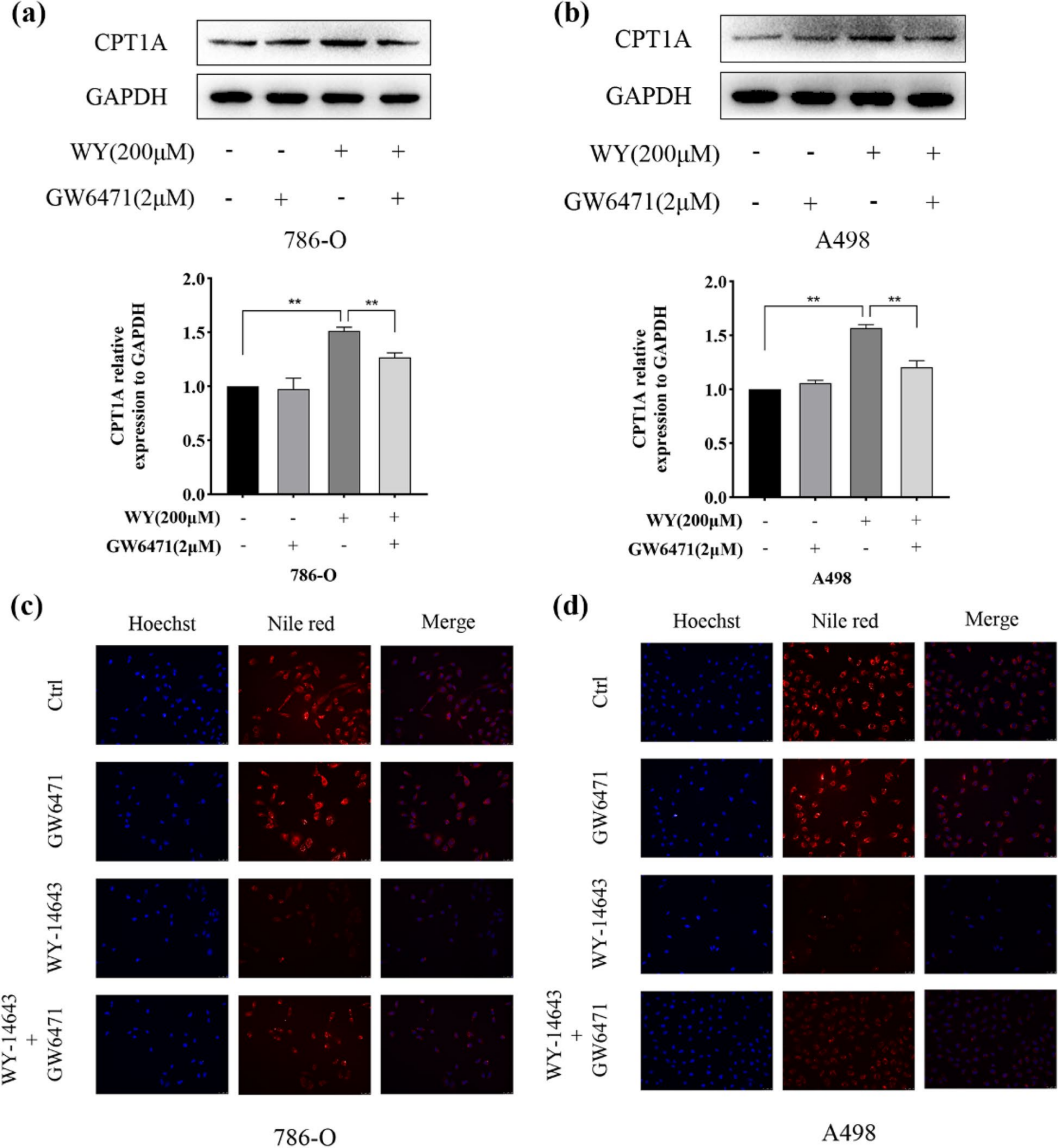
WY-14,643 reduces lipid deposition via the PPARα/CPT1A axis. **a** and **b**. ccRCC cells were treated with GW6471, WY-14,643, or WY-14,643 plus GW6471, and CPT1A protein levels were detected and analyzed quantitatively (*n* = 3). **c** and **d**. Neutral lipid levels were evaluated by Nile red staining after 48 h-treatment with GW6471, WY-14,643, or WY-14,643 plus GW6471 in ccRCC cells, and images were captured. Magnification, 200x. *P < 0.05, **P < 0.01. WY, WY-14,643

As discussed above, WY-14,643 treatment upregulated CPT1A. It has been confirmed that the upregulation of PPARα significantly promotes CPT1A expression and subsequently promotes FAO [31]. In addition, enhanced FAO mediated by PPARα upregulation and WY-14,643 administration was less pronounced in the presence of etomoxir, an irreversible inhibitor of CPT1A [33, 34]. Therefore, the PPARα/CPT1A axis was considered to play a role in WY-14,643-mediated lipid consumption in ccRCC. Then, the function of PPARα in the mechanism by which WY-14,643 upregulates CPT1A was examined by using the PPARα-selective antagonist GW6471 [35]. The treatment concentration of WY-14,643 was set at 200 µM, which is approximately the IC50, to make the results more credible. Moreover, in a cell-based assay, 2 µM GW6471 mostly inhibited the activation of PPARα mediated by 200 µM WY-14,643 [36]. As shown in Fig. 7a and b, GW6471 did not appear to have any obvious effects on CPT1A protein levels. However, CPT1A protein levels were markedly inhibited by WY-14,643 combined with GW6471 in ccRCC cells compared with treatment with WY-14,643 alone. Afterward, Nile red staining was conducted again to assess lipid accumulation. The data in Fig. 7c show that GW6471 did not obviously affect lipid accumulation; nevertheless, treatment with WY-14,643 plus GW6471 in 786-O cells markedly restored lipid accumulation compared with treatment with WY-14,643 alone, and the results were similar in A498 cells (Fig. 7d). After the application of the PPARα inhibitor GW6471, there were synchronous changes in lipid droplets and CPT1A expression levels suggesting a critical role for CPT1A in WY-14,643-mediated lipid consumption. In summary, WY-14,643 treatment reduces lipid deposition via the PPARα/CPT1A axis in ccRCC cells.

### WY-14,643 inhibits the NF-κB pathway in ccRCC cells

**Fig. 8:**
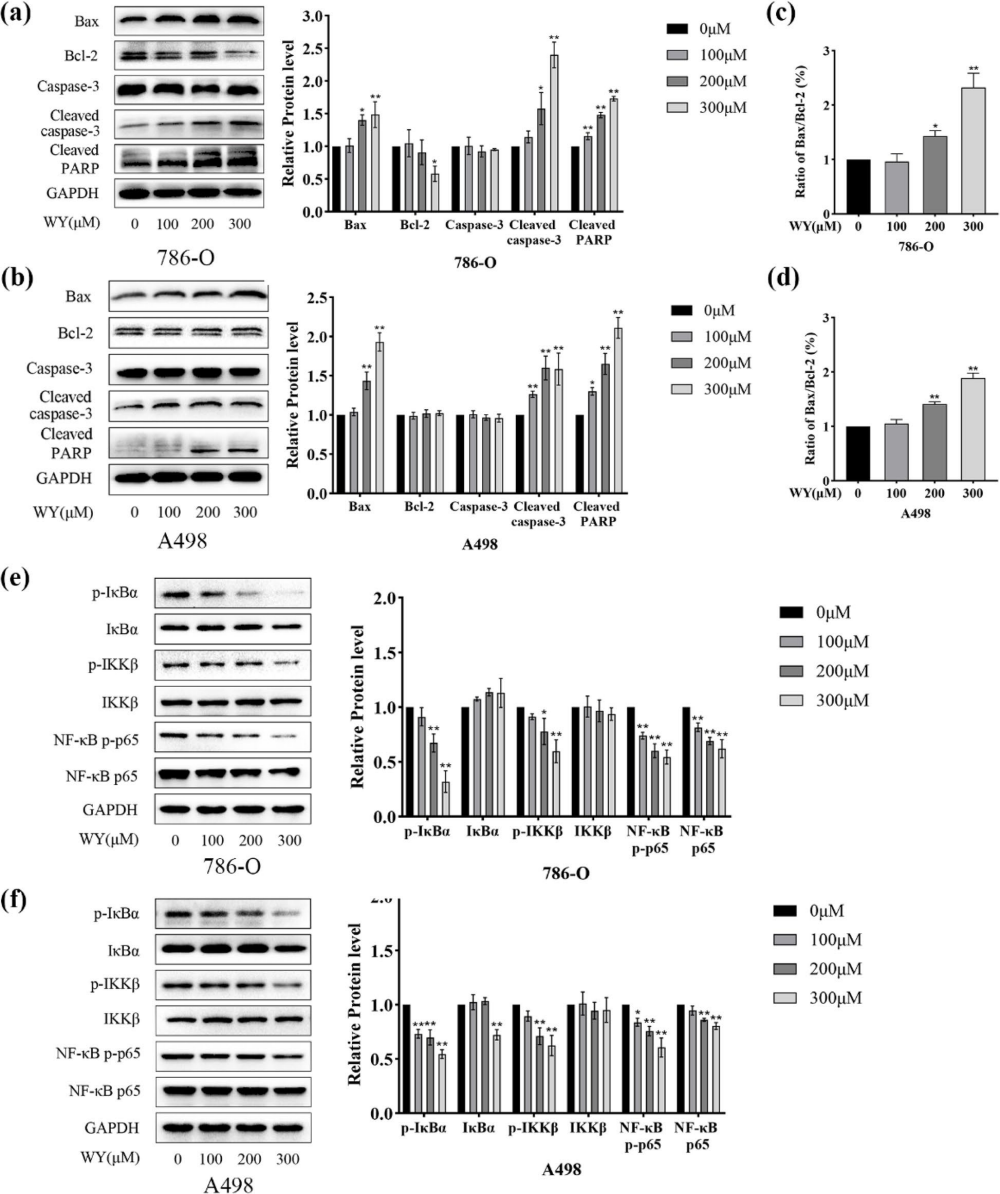
WY-14,643 inhibits the NF-κB pathway. **a** and **b**. After different doses of WY-14,643 were applied, apoptosis-related proteins were evaluated and analyzed quantitatively in ccRCC cells (*n* = 3). **c** and **d**. Bax/Bcl-2 was calculated in ccRCC cells. **e** and **f**. After specified doses of WY-14,643 were administered, the NF-κB pathway were measured by western blotting and analyzed quantitatively (*n* = 3). *P < 0.05, **P < 0.01. WY, WY-14,643

As KEGG and GO enrichment analyses showed that the apoptosis pathway was activated after WY-14,643 administration (Supplementary Fig. 1a and b), apoptosis-related proteins were then detected. As shown in Fig. 8a-d, cleaved caspase-3 and cleaved PARP levels were found to be upregulated, as well as an increase in the ratio of Bax/Bcl-2 after WY-14,643 was administered. It was found that the NF-κB pathway participates in lipid metabolism other than inflammation [37, 38], which can also be inhibited by PPARα via suppressing the phosphorylation of IκBα[39]. To further investigate the mechanism by which WY-14,643 functions, WY-14,643 was added to ccRCC cells, and then, the NF-κB signaling was examined. Figure 8e shows that WY-14,643 significantly suppressed the phosphorylation level of IKKβ and the phosphorylation of IκBα. Moreover, WY-14,643 substantially reduced the expression and the phosphorylation levels of p65, further inhibiting transcriptional regulation in 786-O cells. Figure 8f shows analogous results in A498 cells. The results above indicate that the NF-κB signaling is effectively inhibited after treatment with WY-14,643 in human ccRCC cells. It has been demonstrated that treatment with WY-14,643 upregulates CPT1A and inhibits the NF-κB signaling in the current research, however, the direct connection between CPT1A and NF-κB signaling remains unclear. Thus, 786-O cells were added with specified doses of pyrrolidine dithiocarbamate (PTDC), an NF-κB inhibitor, the applied concentration was based on a previous report [40]. As shown in Supplementary Fig. 2, CPT1A was significantly upregulated after PDTC treatment. In summary, the NF-κB pathway benefits CPT1A upregulation caused by WY-14,643 treatment.

### WY-14,643 activates PPARα/CPT1A by targeting Gly335

**Fig. 9:**
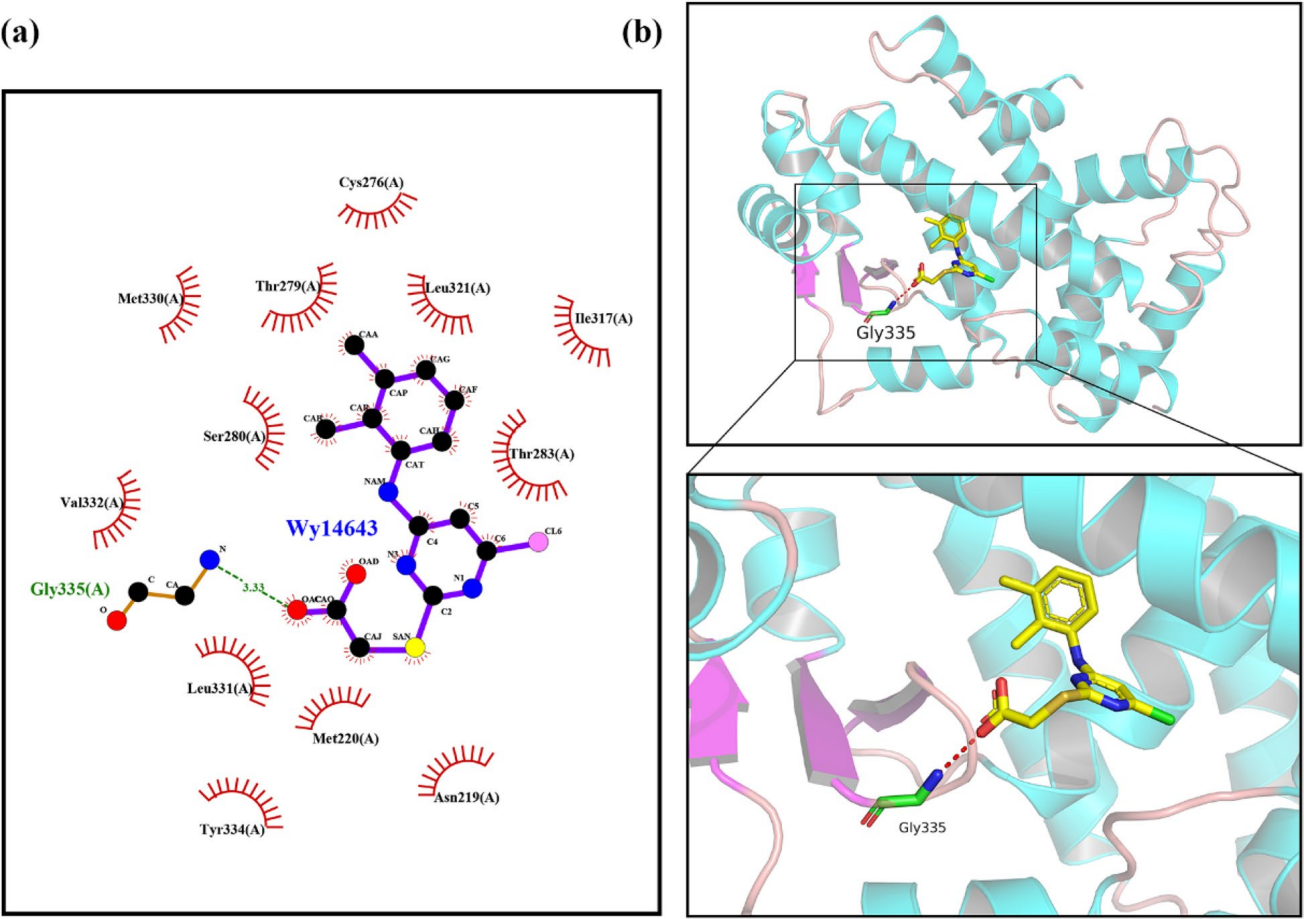
The highest ranked position of WY-14,643 docking the PPARα protein complex. **a**. Interactions between WY-14,643 and the activating residues of PPARα in a 2D model. **b**. Interactions between WY-14,643 and PPARα are delineated in a 3D model. Hydrogen bonds are indicated by red dashed lines, and the docked amino acid residue is also highlighted

The results above have shown that WY-14,643 regulates lipid metabolism in ccRCC by activating PPARα, which is consistent with previous research. To model the interaction between WY-14,643 and the PPARα protein complex, a molecular modeling assay was conducted. As Fig. 9a and b represent, the ligand WY-14,643 enters the protein cavity and binds with the amino acid residue Gly335 of the protein receptor PPARα by forming a 3.33 Å hydrogen bond; at the same time, hydrophobic interactions were formed between the ligand WY-14,643 and other amino acid residues, as shown in Fig. 9a. These hydrogen bonds and hydrophobic interactions make the protein and ligand molecules bind tightly and exert certain physiological activities.

## Discussion

Metastatic ccRCC patients show a poor prognosis, and their quality of life is severely impaired [4]. Accumulating evidence has revealed that ccRCC is an established example of a cancer type undergoing a reprogramming of lipid metabolism during its progression [10]. Repression of fatty acid oxidation driven by HIFs forces fatty acids to be stored in lipid droplets in clear cytoplasm in ccRCC cells [30]; at the same time, lipidomic-associated transcriptomic analysis revealed a higher level of triacylglycerols, phospholipids, and cholesterol esters in ccRCC cancerous tissues than in the surrounding renal cortex [41]. The critical role of CPT1A in abnormal lipid deposition in ccRCC has recently been demonstrated [28, 30]. Targeting abnormal lipid biosynthesis might offer new treatment options for ccRCC [12].

**Fig. 10:**
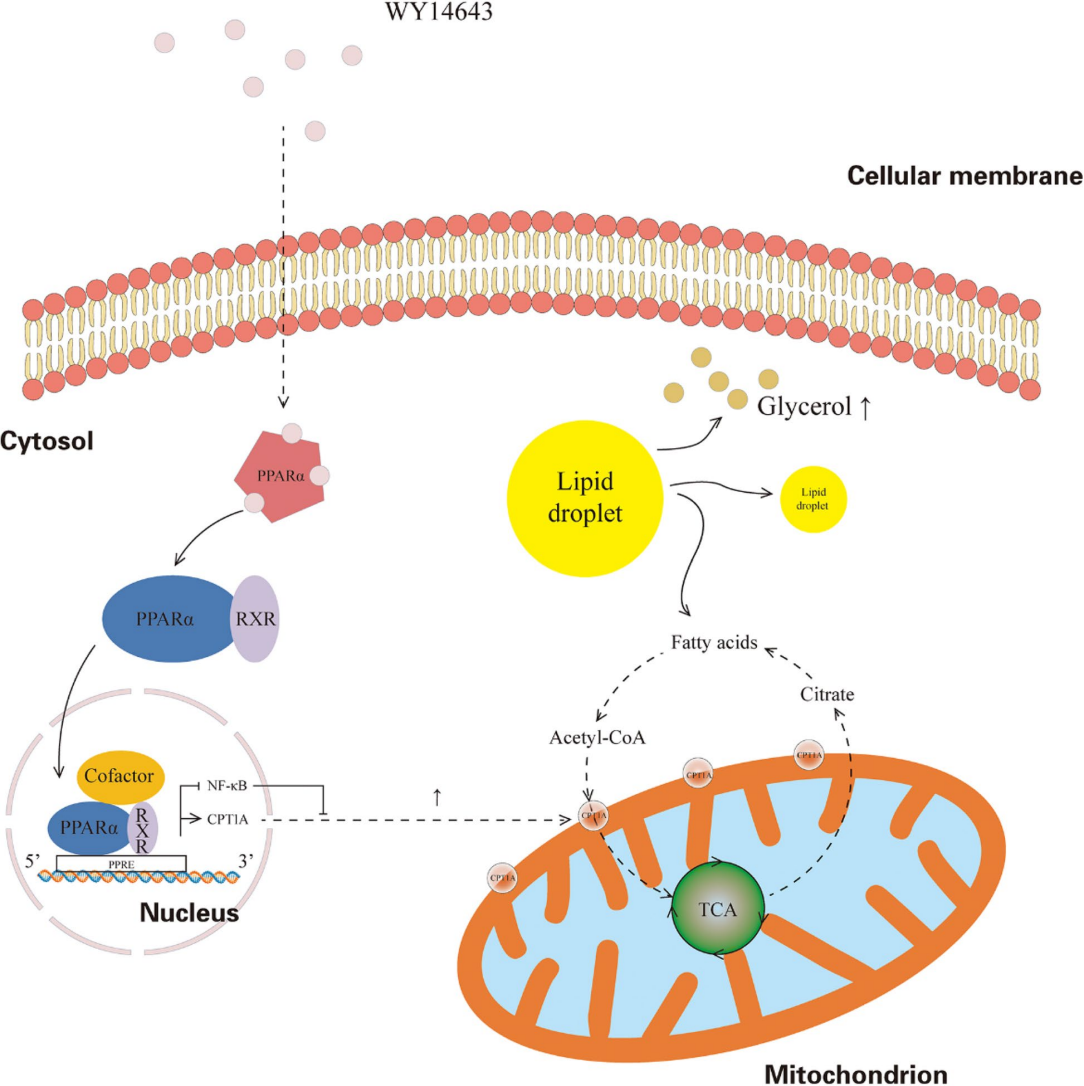
Schematic illustration of the activity of WY-14,643 in ccRCC. WY-14,643 upregulates CPT1A by recognizing and activating PPARα and inhibiting the NF-κB signaling, and then promotes FAO in the mitochondrion, followed by decomposition of lipid droplets

It was demonstrated that WY-14,643 inhibited ccRCC cell proliferation, suppressed ccRCC cell migration, and induced S phase arrest in the present study. Furthermore, it was shown that WY-14,643 attenuated lipid deposition via the PPARα/CPT1A axis, in the process, the NF-κB signaling was also suppressed. Moreover, it was demonstrated by molecular modeling that WY-14,643 activates PPARα by targeting Gly335. The proposed mechanism is detailed in Fig. 10.

WY-14,643 was first utilized as a lipid-lowering drug, and its antagonism effect on PPARα was found to be esponsible for this function soon after [42]. PPARα, a ligand-activated transcription factor, regulates numerous genes responsible for lipid and lipoprotein metabolism [43]. Recently, WY-14,643 has been proven to suppress tumorigenesis in colon cancer and lung cancer in vivo in a PPARα-dependent manner [44]. Considering abnormal lipid accumulation in ccRCC, in this study, WY-14,643 was administered to ccRCC cells. Although 786-O r cells did not show any significant effects on viability after treatment with WY-14,643 at concentrations less than 100 µM in a previous report [45], this is not inconsistent with the above results. In the current study, in ccRCC cells, cell viability was slightly affected after 100 µM WY-14,643 was applied, while 200 µM WY-14,643 treatment exerted an obvious antiproliferative effect. In addition, the purity of the drug might also be a factor.

Inhibition of PPARα/γ by pharmaceutical means could induce EMT through PPAR signaling [46], which might explain that WY-14,643 suppressed ccRCC cell migration in the present study. Most cancer cells gradually acquire mesenchymal features with diminished epithelial characteristics via protein internalization, leading to a ‘partial EMT’ phenotype, which enhances the possibility of tumor metastasis, including ccRCC [32, 47]. In recent studies, metabolic shifts, including lipid metabolism reprogramming, were reported to regulate EMT progression and increase tumor aggressiveness [48]. Furthermore, the induction of EMT was negatively affected by FAO [49, 50]. All these data suggest that the inhibition of EMT by WY-14,643 might partly be ascribed to PPARα-mediated upregulation of FAO.

The DEGs after treatment with WY-14,643 were identified by transcriptomic sequencing. KEGG pathway and GO enrichment analyses showed that multiple canonical pathways were enriched, including the p53 pathway, Hippo signaling, apoptosis and retinol metabolism. These results suggested that WY-14,643 interfered with many biological or cellular processes, including development and differentiation, signal transduction, movement, metabolism, cell communication and response to stress and chemicals. These results also imply that, as a promising treatment for ccRCC, WY-14,643 is worthy of exploration due to its broad effects. Then, the maximum proportion of genes was subjected to metabolism pathways or metabolic processes according to KEGG and GO annotation, indicating that WY-14,643 interferes with metabolism processes in ccRCC, as expected; these results suggest that WY-14,643 participates in metabolic regulation in ccRCC. Therefore, the role of WY-14,643, revealed as a lipid-lowering agent, in lipid metabolism in ccRCC was next investigated in detail.

Growing evidence suggests that PPARα agonism by WY-14,643 is involved in lipid metabolism in multiple organs in addition to the liver [14, 51]. In renal failure, activation of PPARα using WY-14,643 can avoid acute tubular necrosis by promoting fatty acid oxidation [52]. Similarly, this study concluded that WY-14,643 reduced lipid accumulation in ccRCC via PPARα. CPT1A, the rate-limiting enzyme for FAO, was demonstrated to be directly regulated by PPARα [53], and inhibition of CPT1A by etomoxir could rescue the improved FAO due to PPARα upregulation and WY-14,643 administration [33, 34]. Another study has shown that CPT1A inhibition promotes intracellular lipid accumulation by suppressing PPARα and then upregulating CD36 in ccRCC [29]. Therefore, the PPARα/CPT1A axis was supposed to be the mechanism by which WY-14,643 attenuated lipid deposition in ccRCC. After administration of WY-14,643, CPT1A was upregulated in the current research, conforming to the lipids consumption, implying that PPARα/CPT1A might account for this phenomenon. PPI analysis evaluated by STRING and gene correlation analysis through UALCAN and GEPIA also provided some evidence. Herein, WY-14,643 treatment obviously upregulated the CPT1A protein levels; the mRNA levels also increased, but the increase was not statistically significant, which may be related to protein decomposition via the ubiquitin‒proteasome pathway or the asynchrony of transcription and translation. After the PPARα antagonist GW6471 treatment, CPT1A expression levels were markedly decreased and lipid accumulation was restored by treatment with WY-14,643 pus GW6471 compared with that with treatment with WY-14,643 alone. In view of the synchronous changes in lipid droplets and CPT1A expression levels, it is reasonable that the PPARα/CPT1A axis participated in the lipid metabolism regulation mediated by WY-14,643. All the results above suggest that WY-14,643 treatment reduces lipid deposition via the PPARα/CPT1A axis.

There is growing recognition that NF-κB signaling take parts in many processes of cancer development in ccRCC [54]. The NF-κB signaling, a canonical pathway in inflammation, connects metabolism with inflammation during these processes [55]. Cytoplasmic lipid accumulation characterizes ccRCC, while lipid deposition provokes slight, chronic inflammation. NF-κB seems to be the culprit in the production of inflammation in the lipid-overloaded environment [8, 56]. The direct involvement of NF-κB in lipid regulation has been further demonstrated [37, 57, 58]. Hence, in this study, NF-κB signaling was examined by western blotting and found to be suppressed after WY-14,643 was administered, which is consistent with previous results [39]. In the current study, in ccRCC cells, CPT1A was upregulated and the NF-κB signaling was inhibited after WY-14,643 treatment. However, the direct connection between CPT1A and the NF-κB signaling keeps ambiguous. In fact, a negative regulatory effect has been proposed between NF-κB and CPT1A [59]. To verify this, inhibition of the NF-κB pathway by PDTC was performed in 786-O cells. It was shown that CPT1A protein expression levels were increased by PDTC treatment, suggesting that the NF-κB pathway benefits CPT1A upregulation caused by WY-14,643 treatment.

### Comparisons with other studies and what does the current worck add to the existing knowledge

The anticancer effects of WY-14,643 via PPARα activation have recently been discovered in tumors [36, 60]. Here, the anticancer effects of PPARα agonism by WY-14,643 were reported in ccRCC for the first time. Application of WY-14,643 at a higher concentration resulted in a more pronounced anticancer effect in ccRCC in comparison with a previous study [45]. In addition, in existing studies on tumors, the functions of PPARα have received little attention; conversely, the current study provides a possible mechanism by which PPARα regulates lipid metabolism and highlights the anticancer effect of WY-14,643 in ccRCC.

### Study strengths and limitations

The study proposed that PPARα is a therapeutic target for ccRCC due to its role in abnormal lipid accumulation. Activation of PPARα by WY-14,643 exerts anticancer effects on ccRCC cells, which also makes WY-14,643 a promising therapeutic strategy for ccRCC. This research, however, is subject to several limitations. The first limitation concerns the stability of WY-14,643 during cell experiments. Second, the present study focused exclusively on lipid metabolism, but glucose and lipid metabolism interact with each other due to the Warburg effect [10]. In further investigations, more detailed components of glycolipid metabolism will be assessed, and metabolomics might be implemented if necessary. Finally, a more detailed mechanism still needs to be elucidated in further explorations due to the current lack of sufficient references.

## Conclusion

WY-14,643 inhibits the biological behaviors of ccRCC in terms of cell proliferation, migration, and cell cycle arrest. Furthermore, WY-14,643 attenuates lipid deposition, which might contribute to its anticancer properties, at least in part; WY-14,643 acts through the PPARα/CPT1A axis by targeting Gly335, as part of the process, NF-κB signaling is also suppressed. The above findings strongly suggest that PPARα might be an important marker for ccRCC and that lipid regulation by targeting PPARα might be a feasible strategy to improve ccRCC prognosis. This study included in vitro pharmacodynamic experiments, providing a reference and basis for in vivo and clinical experiments of PPARα agonists, including WY-14,643, in the future.

## Supplementary information

**Additional file 1:** **Supplementary Fig. 1.** WY-14643 treatment disrupts lipid metabolism in ccRCC cells. a. pathway enrichment analyses of the DEGs. b. GO enrichment analyses of the DEGs. c. A Heatmap of DEGs in lipid metabolism, including fatty acid binding and activation, fatty acid oxidation, triglyceride turnover, etc. The horizontal axis in a and b represents the enrichment factor. WY, WY-14643.

**Additional file 2:** **Supplementary Fig. 2.** Inhibition of the NF-κB pathway upregulates CPT1A. a. Specified doses of PDTC, a NF-κB inhibitor, were administered to 786-O cells for 24 h, and CPT1A protein expression levels were detected by western blotting and analyzed quantitatively. *P<0.05, **P<0.01.

## Data Availability

All data generated or analyzed during this study are included in this published article.

## Abbreviations

RCC: Renal cell carcinoma
ccRCC: Clear cell renal cell carcinoma
VHL: Von Hippel‒Lindau
HIF: Hypoxia-inducible factor
PPARα: Peroxisome proliferator-activated receptor-α
CPT1A: Carnitine palmitoyltransferase 1A
FAO: Fatty acid oxidation; DMSO: dimethyl sulfoxide
GO: Gene Ontology
KEGG: Kyoto Encyclopedia of Genes and Genomes
DEGs: Differentially expressed genes
PPI: Protein‒protein interaction
STRING: Search Tool for the Retrieval of Interacting Genes
GEPIA: Gene Expression Profiling Interactive Analysis
UALCAN: The University of Alabama at Birmingham Cancer Data Analysis Portal
IC50: Half maximal inhibitory concentration
EMT: Epithelial-mesenchymal transition
PDTC: Pyrrolidine dithiocarbamate

## References

[1] Bray F, Ferlay J, Soerjomataram I, Siegel RL, Torre LA, Jemal A (2018). Global cancer statistics 2018: GLOBOCAN estimates of incidence and mortality worldwide for 36 cancers in 185 countries. Cancer J Clin.

[2] Capitanio U, Bensalah K, Bex A, Boorjian SA, Bray F, Coleman J (2019). Epidemiol Ren Cell Carcinoma Eur Urol.

[3] Díaz-Montero CM, Rini BI, Finke JH (2020). The immunology of renal cell carcinoma. Nat Rev Nephrol.

[4] Jonasch E, Walker CL, Rathmell WK (2021). Clear cell renal cell carcinoma ontogeny and mechanisms of lethality. Nat Rev Nephrol.

[5] Siegel RL, Miller  KD, Jemal A (2020). Cancer statistics. 2020. CA Cancer J Clin.

[6] Park JH, Pyun WY, Park HW (2020). Cancer metabolism: phenotype, signaling and therapeutic targets. Cells..

[7] Cheng C, Geng F, Cheng X, Guo D (2018). Lipid metabolism reprogramming and its potential targets in cancer. Cancer Commun (London England).

[8] Hakimi AA, Reznik E, Lee CH, Creighton CJ, Brannon AR, Luna A (2016). An Integrated Metabolic Atlas of Clear Cell Renal Cell Carcinoma. Cancer Cell.

[9] Chakraborty S, Balan M, Sabarwal A, Choueiri TK, Pal S (2021). Metabolic reprogramming in renal cancer: Events of a metabolic disease. Biochim et Biophys acta Reviews cancer.

[10] Yong C, Stewart GD, Frezza C (2020). Oncometabolites in renal cancer. Nat Rev Nephrol.

[11] Qiu B, Ackerman D, Sanchez DJ, Li B, Ochocki JD, Grazioli A (2015). HIF2α-Dependent Lipid Storage Promotes Endoplasmic Reticulum Homeostasis in Clear-Cell Renal Cell Carcinoma. Cancer Discov.

[12] Wettersten HI, Hakimi AA, Morin D, Bianchi C, Johnstone ME, Donohoe DR (2015). Grade-Dependent Metabolic Reprogramming in Kidney Cancer Revealed by Combined Proteomics and Metabolomics Analysis. Cancer Res.

[13] Santilli AA, Scotese AC, Tomarelli RM (1974). A potent antihypercholesterolemic agent: (4-chloro-6-(2,3-xylidino)-2-pyrimidinylthio) acetic acid (Wy-14643). Experientia.

[14] Li G, Brocker CN, Xie C, Yan T, Noguchi A, Krausz KW (2018). Hepatic peroxisome proliferator-activated receptor alpha mediates the major metabolic effects of Wy-14643. J Gastroenterol Hepatol.

[15] Evans RM, Barish GD, Wang YX (2004). PPARs and the complex journey to obesity. Nat Med.

[16] Porcuna J, Mínguez-Martínez J, Ricote M (2021). The PPARα and PPARγ epigenetic landscape in cancer and immune and metabolic disorders. Int J Mol Sci..

[17] Font-Díaz J, Jiménez-Panizo A, Caelles C, Vivanco MD, Pérez P, Aranda A (2021). Nuclear receptors: Lipid and hormone sensors with essential roles in the control of cancer development. Sem Cancer Biol.

[18] Wu L, Wang W, Dai M, Li H, Chen C, Wang D (2019). PPARα ligand, AVE8134, and cyclooxygenase inhibitor therapy synergistically suppress lung cancer growth and metastasis. BMC Cancer.

[19] Fidoamore A, Cristiano L, Laezza C, Galzio R, Benedetti E, Cinque B (2017). Energy metabolism in glioblastoma stem cells: PPARα a metabolic adaptor to intratumoral microenvironment. Oncotarget.

[20] Luo Y, Xie C, Brocker CN, Fan J, Wu X, Feng L (2019). Intestinal PPARα Protects Against Colon Carcinogenesis via Regulation of Methyltransferases DNMT1 and PRMT6. Gastroenterology.

[21] Cui M, Xiao Z, Wang Y, Zheng M, Song T, Cai X (2015). Long noncoding RNA HULC modulates abnormal lipid metabolism in hepatoma cells through an miR-9-mediated RXRA signaling pathway. Cancer Res.

[22] Wang WL, Welsh J, Tenniswood M (2013). 1,25-Dihydroxyvitamin D3 modulates lipid metabolism in prostate cancer cells through miRNA mediated regulation of PPARA. J Steroid Biochem Mol Biol.

[23] Melone MAB, Valentino A, Margarucci S, Galderisi U, Giordano A, Peluso G (2018). The carnitine system and cancer metabolic plasticity. Cell Death Dis.

[24] Qu Q, Zeng F, Liu X, Wang QJ, Deng F (2016). Fatty acid oxidation and carnitine palmitoyltransferase I: emerging therapeutic targets in cancer. Cell Death Dis.

[25] Wang YN, Zeng ZL, Lu J, Wang Y, Liu ZX, He MM (2018). CPT1A-mediated fatty acid oxidation promotes colorectal cancer cell metastasis by inhibiting anoikis. Oncogene.

[26] Han S, Wei R, Zhang X, Jiang N, Fan M, Huang JH (2019). CPT1A/2-Mediated FAO Enhancement-A Metabolic Target in Radioresistant Breast Cancer. Front Oncol.

[27] Tan Z, Xiao L, Tang M, Bai F, Li J, Li L (2018). Targeting CPT1A-mediated fatty acid oxidation sensitizes nasopharyngeal carcinoma to radiation therapy. Theranostics.

[28] Tan SK, Welford SM (2020). Lipid in Renal Carcinoma: Queen Bee to Target?. Trends Cancer.

[29] Yang H, Zhao H, Ren Z, Yi X, Zhang Q, Yang Z (2022). Overexpression CPT1A reduces lipid accumulation via PPARα/CD36 axis to suppress the cell proliferation in ccRCC. Acta Biochim Biophys Sin.

[30] Du W, Zhang L, Brett-Morris A, Aguila B, Kerner J, Hoppel CL (2017). HIF drives lipid deposition and cancer in ccRCC via repression of fatty acid metabolism. Nat Commun.

[31] Aloia A, Müllhaupt D, Chabbert CD, Eberhart T, Flückiger-Mangual S, Vukolic A (2019). A Fatty Acid Oxidation-dependent Metabolic Shift Regulates the Adaptation of BRAF-mutated Melanoma to MAPK Inhibitors. Clin cancer research: official J Am Association Cancer Res.

[32] Aiello NM, Maddipati R, Norgard RJ, Balli D, Li J, Yuan S (2018). EMT Subtype Influences Epithelial Plasticity and Mode of Cell Migration. Dev Cell.

[33] Li Y, Xiong Z, Yan W, Gao E, Cheng H, Wu G (2020). Branched chain amino acids exacerbate myocardial ischemia/reperfusion vulnerability via enhancing GCN2/ATF6/PPAR-α pathway-dependent fatty acid oxidation. Theranostics.

[34] Knight BL, Hebbachi A, Hauton D, Brown AM, Wiggins D, Patel DD (2005). A role for PPARalpha in the control of SREBP activity and lipid synthesis in the liver. Biochem J.

[35] Xu HE, Stanley TB, Montana VG, Lambert MH, Shearer BG, Cobb JE (2002). Structural basis for antagonist-mediated recruitment of nuclear co-repressors by PPARalpha. Nature.

[36] Hwang YP, Won SS, Jin SW, Lee GH, Pham TH, Choi JH (2019). WY-14643 Regulates CYP1B1 expression through peroxisome proliferator-activated receptor α-mediated signaling in human breast cancer cells. Int J Mol Sci..

[37] Yu XH, Zheng XL, Tang CK (2015). Nuclear Factor-κB Activation as a Pathological Mechanism of Lipid Metabolism and Atherosclerosis. Adv Clin Chem.

[38] Wu HM, Ni XX, Xu QY, Wang Q, Li XY, Hua J (2020). Regulation of lipid-induced macrophage polarization through modulating peroxisome proliferator-activated receptor-gamma activity affects hepatic lipid metabolism via a Toll-like receptor 4/NF-κB signaling pathway. J Gastroenterol Hepatol.

[39] Huang D, Zhao Q, Liu H, Guo Y, Xu H, PPAR-α Agonist (2016). WY-14643 Inhibits LPS-Induced Inflammation in Synovial Fibroblasts via NF-kB Pathway. J Mol neuroscience: MN.

[40] Morais C, Pat B, Gobe G, Johnson DW, Healy H (2006). Pyrrolidine dithiocarbamate exerts anti-proliferative and pro-apoptotic effects in renal cell carcinoma cell lines. Nephrology, dialysis, transplantation: official publication of the European Dialysis and Transplant Association. Eur Ren Association.

[41] Saito K, Arai E, Maekawa K, Ishikawa M, Fujimoto H, Taguchi R (2016). Lipidomic Signatures and Associated Transcriptomic Profiles of Clear Cell Renal Cell Carcinoma. Sci Rep.

[42] Pollinger J, Merk D (2017). Therapeutic applications of the versatile fatty acid mimetic WY14643. Expert opinion on therapeutic patents..

[43] Yoon M (2009). The role of PPARalpha in lipid metabolism and obesity: focusing on the effects of estrogen on PPARalpha actions. Pharmacol Res.

[44] Pozzi A, Ibanez MR, Gatica AE, Yang S, Wei S, Mei S (2007). Peroxisomal proliferator-activated receptor-alpha-dependent inhibition of endothelial cell proliferation and tumorigenesis. J Biol Chem.

[45] Mao R, Shi J, Ma X, Xu H (2021). Hydroxychloroquine Potentiates Apoptosis Induced by PPARα Antagonist in 786-O Clear Cell Renal Cell Carcinoma Cells Associated with Inhibiting Autophagy. PPAR Res.

[46] Wu CT, Wang CC, Huang LC, Liu SH, Chiang CK (2018). Plasticizer Di-(2-Ethylhexyl)Phthalate Induces Epithelial-to-Mesenchymal Transition and Renal Fibrosis In Vitro and In Vivo. Toxicol Sci.

[47] Piva F, Giulietti M, Santoni M, Occhipinti G, Scarpelli M, Lopez-Beltran A (2016). Epithelial to Mesenchymal Transition in Renal Cell Carcinoma: Implications for Cancer Therapy. Mol Diagn Ther.

[48] Kang H, Kim H, Lee S, Youn H, Youn B (2019). Role of Metabolic reprogramming in epithelial–Mesenchymal Transition (EMT). Int J Mol Sci..

[49] Dalmau N, Jaumot J, Tauler R, Bedia C (2015). Epithelial-to-mesenchymal transition involves triacylglycerol accumulation in DU145 prostate cancer cells. Mol Biosyst.

[50] Yang L, Zhang F, Wang X, Tsai Y, Chuang KH, Keng PC (2016). A FASN-TGF-β1-FASN regulatory loop contributes to high EMT/metastatic potential of cisplatin-resistant non-small cell lung cancer. Oncotarget.

[51] Yan J, Song K, Bai Z, Ge RL (2021). WY14643 improves left ventricular myocardial mitochondrial and systolic functions in obese rats under chronic persistent hypoxia via the PPARα pathway. Life Sci.

[52] Li S, Wu P, Yarlagadda P, Vadjunec NM, Proia AD, Harris RA (2004). PPAR alpha ligand protects during cisplatin-induced acute renal failure by preventing inhibition of renal FAO and PDC activity. Am J Physiol Ren Physiol.

[53] Honda K, Saneyasu T, Sugimoto H, Kurachi K, Takagi S, Kamisoyama H (2016). Role of peroxisome proliferator-activated receptor alpha in the expression of hepatic fatty acid oxidation-related genes in chickens. Anim Sci journal = Nihon chikusan Gakkaiho.

[54] Morais C, Gobe G, Johnson DW, Healy H (2011). The emerging role of nuclear factor kappa B in renal cell carcinoma. Int J Biochem Cell Biol.

[55] Tornatore L, Thotakura AK, Bennett J, Moretti M, Franzoso G (2012). The nuclear factor kappa B signaling pathway: integrating metabolism with inflammation. Trends Cell Biol.

[56] Kauppinen A, Suuronen T, Ojala J, Kaarniranta K, Salminen A (2013). Antagonistic crosstalk between NF-κB and SIRT1 in the regulation of inflammation and metabolic disorders. Cell Signal.

[57] Heida A, Gruben N, Catrysse L, Koehorst M, Koster M, Kloosterhuis NJ (2021). The hepatocyte IKK:NF-κB axis promotes liver steatosis by stimulating de novo lipogenesis and cholesterol synthesis. Mol metabolism.

[58] Daniel PV, Dogra S, Rawat P, Choubey A, Khan AS, Rajak S (2021). NF-κB p65 regulates hepatic lipogenesis by promoting nuclear entry of ChREBP in response to a high carbohydrate diet. J Biol Chem.

[59] Tayyeb JZ, Popeijus HE, Mensink RP, Konings M, Mokhtar FBA, Plat J (2020). Short-chain fatty acids (Except Hexanoic Acid) Lower NF-kB Transactivation, which rescues Inflammation-induced decreased Apolipoprotein A-I transcription in HepG2 Cells. Int J Mol Sci..

[60] Skrypnyk N, Chen X, Hu W, Su Y, Mont S, Yang S (2014). PPARα activation can help prevent and treat non-small cell lung cancer. Cancer Res.

