# Supplementary material for: WY-14643 attenuates lipid deposition via activation of the PPARα/CPT1A axis by targeting Gly335 to inhibit cell proliferation and migration in ccRCC
Source: Lipids Health Dis. 2022 Nov 16;21:121. doi: 10.1186/s12944-022-01726-7 (PMC9667690; doi:10.1186/s12944-022-01726-7)
Supplement: Supplementary file 2 — Additional file 2: Supplementary Fig. 2. Inhibition of the NF-κB pathway upregulates CPT1A. a. Specified doses of PDTC, a NF-κB inhibitor, were administered to 786-O cells for 24 h, and CPT1A protein expression levels were detected by western blotting and analyzed quantitatively. *P<0.05, **P<0.01. [file 12944_2022_1726_MOESM2_ESM.pdf]

Editing Certificate

This document certifies that the manuscript

WY-14643 attenuates lipid deposition via activation of the PPARα/CPT1A axis by targeting Gly335 to inhibit cell proliferation and migration in ccRCC

prepared by the authors

Rui Wang, Jun Zhao

was edited for proper English language, grammar, punctuation, spelling, and overall style by one or more of the highly qualified native English speaking editors at AJE.

This certificate was issued on **October 5, 2022** and may be verified on the AJE website using the verification code **F49C-8CF0-B6CC-8EFE-C3FP**.

Neither the research content nor the authors' intentions were altered in any way during the editing process. Documents receiving this certification should be English-ready for publication; however, the author has the ability to accept or reject our suggestions and changes. To verify the final AJE edited version, please visit our verification page at aje.com/certificate. If you have any questions or concerns about this edited document, please contact AJE at support@aje.com.

AJE provides a range of editing, translation, and manuscript services for researchers and publishers around the world. For more information about our company, services, and partner discounts, please visit aje.com.
